# Selective Separation of Singly Charged Chloride and Dihydrogen Phosphate Anions by Electrobaromembrane Method with Nanoporous Membranes

**DOI:** 10.3390/membranes13050455

**Published:** 2023-04-23

**Authors:** Dmitrii Butylskii, Vasiliy Troitskiy, Daria Chuprynina, Ivan Kharchenko, Ilya Ryzhkov, Pavel Apel, Natalia Pismenskaya, Victor Nikonenko

**Affiliations:** 1 Membrane Institute, Kuban State University, 149 Stavropolskaya St., 350040 Krasnodar, Russia; 2 Department of Analytical Chemistry, Kuban State University, 149 Stavropolskaya St., 350040 Krasnodar, Russia; 3 Institute of Computational Modeling SB RAS, 50-44 Akademgorodok, 660036 Krasnoyarsk, Russia; 4 Siberian Federal University, 79 Svobodny, 660041 Krasnoyarsk, Russia; 5 Joint Institute for Nuclear Research, 6 Joliot-Curie St., 141980 Dubna, Russia

**Keywords:** ion separation, highly selective separation, phosphate, electrobaromembrane separation, countercurrent electromigration, nanoporous membranes

## Abstract

The entrance of even a small amount of phosphorus compounds into natural waters leads to global problems that require the use of modern purification technologies. This paper presents the results of testing a hybrid electrobaromembrane (EBM) method for the selective separation of Cl^−^ (always present in phosphorus-containing waters) and H_2_PO_4_^−^ anions. Separated ions of the same charge sign move in an electric field through the pores of a nanoporous membrane to the corresponding electrode, while a commensurate counter-convective flow in the pores is created by a pressure drop across the membrane. It has been shown that EBM technology provides high fluxes of ions being separated across the membrane as well as a high selectivity coefficient compared to other membrane methods. During the processing of solution containing 0.05 M NaCl and 0.05 M NaH_2_PO_4_, the flux of phosphates through a track-etched membrane can reach 0.29 mol/(m^2^×h). Another possibility for separation is the EBM extraction of chlorides from the solution. Its flux can reach 0.40 mol/(m^2^×h) through the track-etched membrane and 0.33 mol/(m^2^×h) through a porous aluminum membrane. The separation efficiency can be very high by using both the porous anodic alumina membrane with positive fixed charges and the track-etched membrane with negative fixed charges due to the possibility of directing the fluxes of separated ions in opposite sides.

## 1. Introduction

The development of new approaches for the extraction of valuable components from aqueous solutions is an important applied problem. A number of recent papers have noted the high potential of membrane methods for the selective extraction of nutrients (biologically significant chemical elements) and inorganic ions (e.g., lithium, cobalt, and nickel) from waste and industrial waters, leachate solution, etc. [1,2,3,4], as well as for reagentless production/recovery of acids and bases [5]. The advantage of membrane methods compared to traditional reagent-based methods is low energy and resource consumption (almost no chemical reagents are required), as well as high environmental friendliness (no additional waste streams). An important fact is also the ability to recover the majority of water for reuse. This reduces the involvement of new water resources and allows creating water recycling systems in production processes [6,7].

Waste and industrial waters are characterized by a variety of chemical composition, which is mainly determined by the source of their entry into the environment. Cations (sodium, potassium, calcium, magnesium, iron, etc.), anions (chlorides, nitrates, phosphates, etc.), dissolved gases (oxygen, nitrogen, etc.), organic compounds, microorganisms, etc., may be dominated in their composition. However, it is the removal of phosphates (phosphorus compounds in the general case) from waste and industrial waters that has acquired the status of a global task and is being addressed involving the political level [4,8,9]. The reason is the fact that the high biogenic activity of phosphorus compounds leads to the eutrophication of natural waters (rapid growth of blue-green algae) even at trace levels (less than 1 ppm).

The main methods for extracting phosphorus from aqueous solutions discussed in the literature and used in industry are anerobic biochemical digestion (AnD), liquid extraction, crystallization, precipitation, adsorption, and ion exchange [10,11,12,13,14,15,16,17,18,19]. Membrane methods for removing phosphorus compounds are under development [2,4,20].

Pressure-driven membrane processes (nanofiltration, NF; reverse and direct osmosis, RO&DO) are used in many phosphorus recovery processes for the separation of substances [4]. For example, commercial NF and RO membranes of various types show excellent retention rates for phosphorus varying mainly in the range of 74–99% [21,22]. The retention rate of phosphorus applied in practice strongly depends on the composition of the processed solution [23]. The desire to increase this parameter is limited by the risk of salt deposition on the surface and in the pores of the membranes [6,24]. In this regard, many researchers are looking for membrane materials (or their modifiers) that are resistant to the deposition [24,25].

Together with the value of the operating pressure and the composition of the processed solution, the pH value of the feed solution also affects the productivity of phosphorus extraction in the pressure-driven membrane process. When the pH value increases, the phosphorus retention rate also increases. This is apparently due to the formation of doubly charged phosphate anions, which are better retained by nanofiltration membranes [26]. At low pH values, acid molecules and a singly charged dihydrogen phosphate anion predominate in the solution, which do not encounter significant resistance to transfer through negatively charged membranes.

For the electrodialysis, ED, and recovery of phosphorus, the pH value of the feed solution also plays an important role. When the pH of the processed solution is in the range of 3.7–6.0, H_2_PO_4_^−^ ions predominate in it. However, not only these anions but also doubly charged HPO_4_^2−^ anions are transported through conventional anion-exchange membranes. The latter is due to the fact that the pH in their pore solution is 1–3 pH units higher than in the feed solution [2,27,28]. In addition, the mechanism of transfer of phosphoric acid ions can be complicated by the reaction of catalytic generation of H^+^ and OH^−^ ions with the participation of fixed groups of the ion-exchange membrane in overlimiting current modes [29,30]. However, despite the complicated mechanism of phosphate species transport, the flux of phosphorus through AEM is determined by electrodiffusion of phosphate anions from the bulk solution to the membrane/depleted diffusion layer (DBL) interface, as in the case of strong electrolyte ions [30].

Usually, an external electric field and a pressure field are used separately in membrane methods. A completely different approach is used in hybrid electrobaromembrane (EBM) methods, when separation occurs under the action of an electric field and pressure applied simultaneously [31,32]. Separated ions of the same charge sign move under the influence of an electric field through the pores of a nanoporous membrane to the corresponding electrode, while a commensurate counter-convective flow is created in the pores by a pressure drop across the membrane. Ion separation is achieved due to the difference in the mobility of the ions being separated [32,33].

This method was first used by Brewer et al. [34,35] in the separation of potassium isotopes. It was later adapted for membrane systems by Konturri et al. [36,37,38,39,40,41]. In recent works, when using polyethylene terephthalate track-etched membranes in EBM devices, it was possible to achieve impressive results in the separation of Li^+^/K^+^ and Li^+^/Na^+^ pairs [31,32,42]. The EBM method was also used to extract ions of weak electrolytes, for example, when separating acetic acid and monochloroacetic acid anions, which differ slightly in mobility in an electric field [37].

Thus, the hybrid EBM method can be effectively used for the separation of singly charged ions of strong (e.g., Li^+^/K^+^ or Li^+^/Na^+^) and weak (acetic acid anions) electrolytes. However, as far as is known, no attempts have been made to apply this method for the separation of phosphate species. In this regard, the purpose of this work is to expand the scope of the EBM method and test it in the separation of Cl^−^ and H_2_PO_4_^−^ ions. The results are obtained using two types of membranes: a polyethylene terephthalate track-etched membrane and a porous anodic alumina membrane.

## 2. Materials and Methods

For a simplified representation of the wastewater composition, a ternary feed solution containing sodium salts of chlorides and phosphates is used. These anions are present in almost all wastewaters, especially in the wastewaters of agriculture and the livestock sector. The main characteristics of the feed solution components that affect the efficiency of EBM separation are given in Table 1.

**Figure 1:**
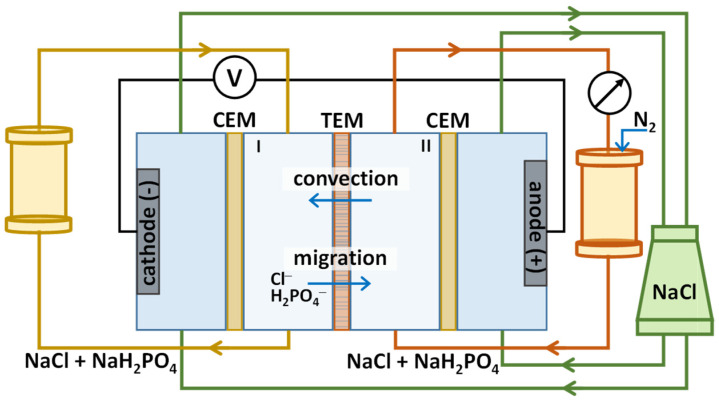
A schematic diagram of the setup for studying the parameters of the selective separation of Cl^−^ and H_2_PO_4_^−^ anions by hybrid electrobaromembrane method.

The design of a four-chamber, flow-through laboratory electrodialysis cell was used to obtain separation parameters using the hybrid EBM method (Figure 1). A solution containing a mixture of 0.05 M NaCl and 0.05 M NaH_2_PO_4_ (pH = 3.8–3.9) was pumped through the left-hand (I) and right-hand (II) chambers, separated by a porous membrane, at the same flow rate (90 mL/min), as it was performed in previous experimental work on the selective separation of Li^+^ and K^+^ [32].

In addition to the porous membrane, chambers I and II are formed using auxiliary cation-exchange (CEM) MK-40 heterogeneous membranes (JCC Shchekinoazot, Pervomayskiy, Russia). These membranes served to prevent the transfer of anions from the cathode chamber to chamber I and their exit from chamber II. A 0.1 M NaCl solution was pumped through the electrode chambers; polarizing platinum electrodes were used. On both sides of the porous membrane, the Luggin capillaries were installed to control the voltage across the membrane. A convective flow was created, directed from chamber II to chamber I, opposite to the migration of competing anions. This was achieved by increasing the pressure of the solution in the circuit passing through chamber II, using an automatic nitrogen dosing system. The composition of solutions in chambers I and II was monitored with a pH meter and a conductometer. The experiment on separation was repeated at least 5 times with the given parameters. Samples of the solution from chambers I and II were taken at the beginning and the end of the separation process to determine the concentration of separated ions using a Dionex ICS-3000 ion chromatograph with the conductometric detector (Thermo Fisher Scientific, Waltham, MA, USA).

**Figure 2:**
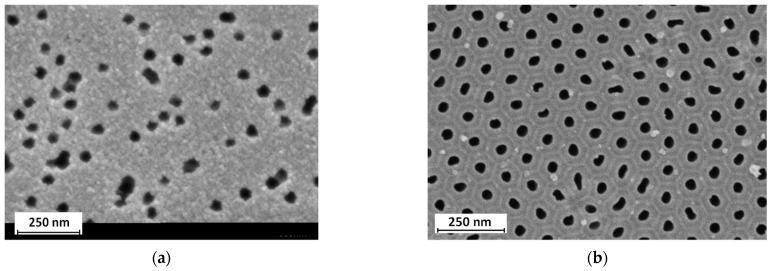
SEM images of porous membrane surfaces used in this study: (**a**) track-etched membrane and (**b**) anodic alumina membrane.

Two types of membranes were used for separating Cl^−^ and H_2_PO_4_^−^ ions: a polyethylene terephthalate track-etched membrane manufactured and labeled as TEM #811 at the Joint Institute for Nuclear Research (Dubna, Russia) and an inorganic porous anodic alumina membrane (PAAM) manufactured at the Krasnoyarsk Scientific Center of the Siberian Branch of the Russian Academy of Sciences (Krasnoyarsk, Russia) (Figure 2).

Both types of membranes have relatively close pore size (Table 2). However, in the case of PAAM, the specific number of pores and thickness determine its main difference from TEM #811.

Preparation of porous anodic alumina membranes

The aluminum foil of high purity (99.999%) with a thickness of 500 µm was used for the membrane preparation [46]. The foil was electrochemically polished in a solution of 1.85 M CrO_3_ and 15.1 M H_3_PO_4_ at 80 °C in pulsed mode. The duration of each pulse was 3 s with a current density of 0.5 A/cm^2^ and the interpulse interval was 40 s. A total of 40 pulses were applied. The anodization was performed in a 0.3 M oxalic acid electrolyte in potentiostatic mode at 40 V. The electrolyte was kept at the temperature of 4 °C with intensive stirring. The anodized area was a circle with a diameter of 20 mm on a 40 mm diameter aluminum foil. After the first anodization for 10 h, the alumina layer was removed in a solution of 0.2 M CrO_3_ and 0.6 M H_3_PO_4_ at 60 °C for 40 min. The second anodization was performed under the same conditions until the total charge density reached 186 C/cm^2^, which resulted in a membrane thickness of 80 µm and an anodization time of around 30 h. The aluminum substrate was selectively etched in the form of 20 mm diameter circles in a solution of 0.25 M CuCl_2_ and 5 vol % HCl. After that, the barrier layer was removed using a solution of 0.5 M H_3_PO_4_ with electrochemical detection of the pore opening [47]. According to SEM images, the average pore diameter in the obtained membranes was 44 ± 2 nm.

Determination of membrane ion separation coefficient

The ion separation coefficient, $S_{1/2}$, (also called the (specific) permselectivity coefficient between two counterions [48]) is used to quantify the selective transport of ions across a membrane. It is defined by the following equation [3,49,50]:

(1) $$S_{1/2}=\frac{j_{1}/j_{2}}{c_{1}^{f}/c_{2}^{f}}=\frac{\Delta c_{1}^{p}/\Delta c_{2}^{p}}{c_{1}^{f}/c_{2}^{f}}=\frac{P_{1},\%}{P_{2},\%}$$

where $j_{k}$ is the flux density of ions *i* through the membrane (where *k* = 1, 2 and corresponds to different types of ions of the same charge sign); $c_{k}^{f}$ is the concentration of ions *i* in the feed solution; $\Delta c_{k}^{p}$ is the change in the concentration of ions *k* in the permeate (in the case of NF) or in the concentrate (in the case of ED); $P_{k}$ is the ion passage (use in pressure-driven membrane process), which is defined as $P_{k}=(c_{k}^{p}/c_{k}^{f})\times 100\%$. Concentrations and fluxes must be expressed in the same units of the amount of substance (mols or equiv.).

To calculate the fluxes of ions *k* through the membrane, the following equation is used:

(2) $$j_{k}=\frac{V}{s}\frac{dc_{k}}{dt}$$

where *V* is the volume of the processed solution or permeate (concentrate), *s* is the effective membrane surface area, and *t* is the duration of the experiment.

## 3. Results

As noted above, the polybasicity of phosphoric acid anions is the main problem for their effective extraction from wastewater by membrane methods. In the process of EBM separation, as in the case of pressure-driven membrane processes, the proportion of the predominant form of the orthophosphoric acid anion does not change with time. On the other hand, the pH of the feed solution can change when the limiting current density reached the auxiliary CEMs that form chambers I and II. This change in pH is caused by proton-transfer catalytic reactions between the fixed groups of the membrane and water molecules at the ion-exchange membrane/desalted solution interface (“water splitting” [29,51]). Water splitting takes place when the concentration of charge carriers in the electrolyte reaches close to zero value at this interface [52]. Upon reaching the limiting state on the auxiliary membranes, the pH value should decrease in chamber II and increase in chamber I. If the pH value decreases in chamber II, then the efficiency of EBM separation will be affected by the diffusion transfer of orthophosphoric acid molecules from chamber II to chamber I through the porous membrane. The appearance of additional current carriers (H^+^/OH^−^ ions) in the EBM system will lead to a decrease in current efficiency due to the transfer of protons and hydroxide ions through the track membrane. In addition, the change in pH will lead to a change in the charge of phosphate acid species. In particular, an increase in pH will result in the appearance of HPO_4_^2−^ anions. The performance of EBM separation would be reduced. In this regard, in each EBM system under study, preliminary tests to determine the limiting current on the auxiliary membranes were carried out. Then underlimiting and close to the limiting current densities were used in order to avoid the development of the described unwanted effects.

With regard to theoretical analysis, it has previously been shown that a simplified model can be used that allows for a better understanding of the principles of the method without claiming quantitative agreement [32]. This model does not provide as much detail as the countercurrent flow separation models based on the Nernst–Planck equations [33,38,53]. However, its simplicity makes it accessible to a wide range of researchers.

For the calculation of flux densities *j_k_* of competing counterions (*k* = 1, 2), the contributions of electromigration, diffusion, and convection (denoted below by subscripts *migr*, *dif*, and *conv*, respectively) were taken into account as follows:

(3) $$j_{1}=j_{1}^{migr}+j_{1}^{dif}+j_{1}^{conv}=\frac{i{\tilde{t}}_{1}}{z_{1}F}+c_{1}v^{conv}\gamma$$

(4) $$j_{2}=j_{2}^{migr}+j_{2}^{dif}+j_{2}^{conv}=\frac{i{\tilde{t}}_{2}}{z_{2}F}+c_{2}v^{conv}\gamma$$

where *i* is the current density (in A/m^2^ of the membrane surface), ${\tilde{t}}_{k}$, *c_k_*, and *z_k_* are the effective transport number (dimensionless), concentration (in mol/m^3^ of the pore solution), and charge number of ion *k*, $v^{conv}$ is the average convective velocity (in *m*/*s*), and γ is the surface porosity.

The first term in Equations (3) and (4) describes the joint contribution of electromigration and diffusion. This is taken into account in the value of the effective transport number, ${\tilde{t}}_{k}$. Thereby, ${\tilde{t}}_{k}$ is the fraction of electric charge carried by ion *k* under the action of electric current and diffusion (if any). The second term in Equations (3) and (4) describes the convection contribution. It does not depend on the set current, but is determined only by the electrolyte concentration in the membrane pores, the membrane porosity, and the flow rate of the electrolyte solution through the membrane. The velocity of pressure-driven flow through a pore of diameter *d* is described by the Hagen–Poiseuille equation:

(5) $$v_{}^{conv}=\frac{1}{32}\frac{\Delta pd^{2}}{\eta L}$$

where ∆*p* is the pressure difference between chamber II and chamber I, *L* is the length of a pore, and *η* is the liquid viscosity.

$v^{conv}$ is linked with the flow rate *W* of solution through the membrane with surface *S*: $W=v^{conv}S\gamma$.

### 3.1. Track-Etched Membrane

**Figure 3:**
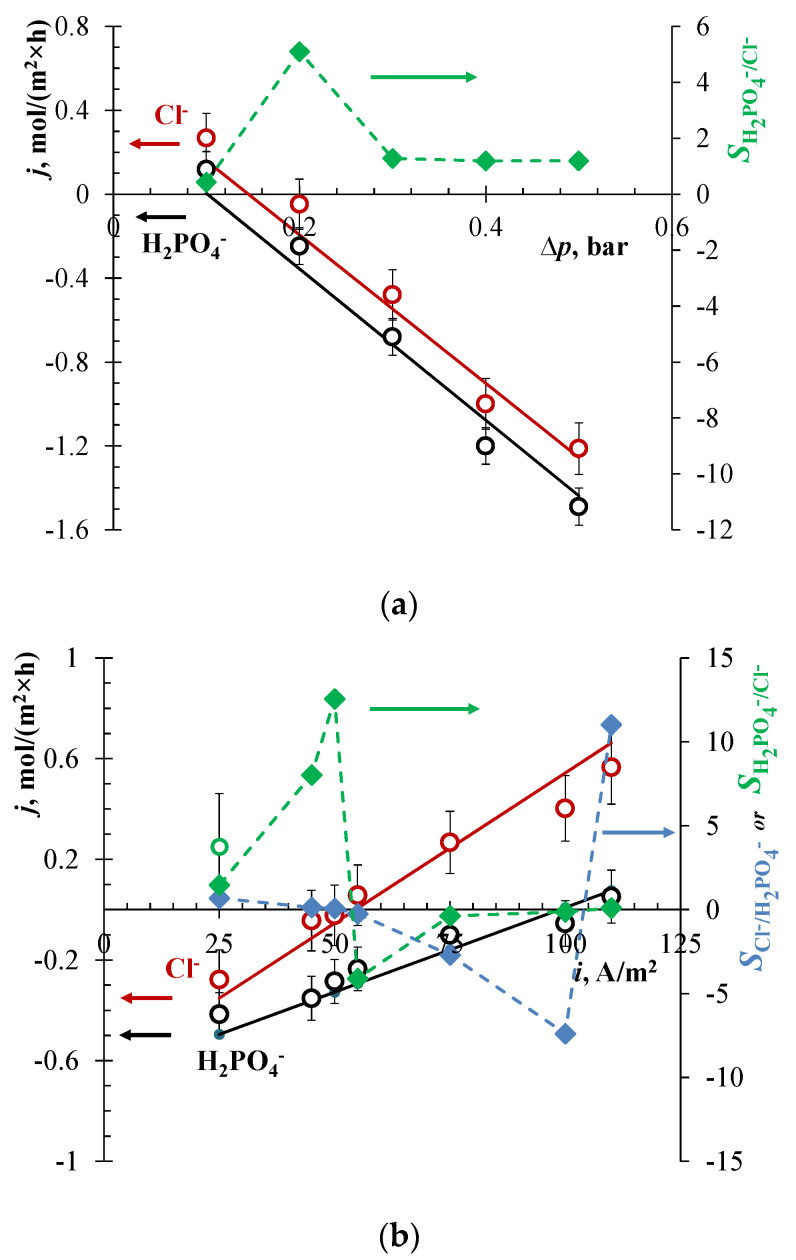
Flux densities of Cl^−^ and H_2_PO_4_^−^ ions (circles) through the TEM #811 membrane, as well as the separation coefficient, *S* (diamonds), vs. pressure difference at a constant current density of 25 A/m^2^ (**a**), and the flux densities of the same ions vs. current density at a constant pressure difference of 0.3 bar (**b**). Experimental data are shown by markers, and solid lines are calculated using Equations (3)–(5) setting *d* = 32 nm, ${\tilde{t}}_{Cl^{-}}$ = 0.55, and ${\tilde{t}}_{H_{2}PO_{4}^{-}}$ = 0.39 (**a**); *d* = 32 nm, ${\tilde{t}}_{Cl^{-}}$ = 0.32, and ${\tilde{t}}_{H_{2}PO_{4}^{-}}$ = 0.18 (**b**) as fitting parameters, as well as the membrane parameters presented in Table 2.

A track-etched membrane labeled as TEM #811 was used earlier for the separation of lithium and potassium by the EBM method [32]. A high separation selectivity was achieved due to a high ratio of the mobilities of these ions. The mobility of chlorides is approximately two times higher than the mobility of dihydrogen phosphate ions. Therefore, a high separation selectivity can be expected for this ion pair also. When a constant current density of 25 A/m^2^ is set in the EBM system, the fluxes of competing ions through the membrane separating chambers I and II at a low pressure drop of 0.1 bar are determined by electromigration: $j_{Cl^{-}}/j_{H_{2}PO_{4}^{-}}\approx j_{Cl^{-}}^{migr}/j_{H_{2}PO_{4}^{-}}^{migr}\approx 2$ (Figure 3a). With an increase in pressure, the fluxes of both ions are slowed down by countercurrent convection and become negative with respect to the transport of ions in an electric field. When the pressure difference is close to 0.2 bar, the flux of Cl^−^ ions is very small, and the oppositely directed migration and convection almost cancel each other out. At the same time, the flux of H_2_PO_4_^−^ ions becomes negative and relatively large in magnitude: for this ion, convection prevails. However, this scheme of the experiment has limitations: It is more difficult to fix an exact pressure drop than to control the current.

When a constant pressure drop of 0.3 bar between chambers I and II is applied, and the current density is relatively low (e.g., 25 A/m^2^, Figure 3b), the convection transport of both chlorides and phosphates dominates over their electromigration transport. Their fluxes are negative, since the electromigration velocity is much lower than the convection velocity.

As in the case of cations [3], there are various options for the selective separation of Cl^−^ and H_2_PO_4_^−^ ions by the EBM method. With an increase in the value of the current, the resulting chloride flux through the track membrane approaches zero: at 50 A/m^2^, $j_{Cl^{-}}$ = −0.022 mol/(m^2^×h) (Figure 3b). However, the phosphate flux is still determined by the convective component, $j_{H_{2}PO_{4}^{-}}$ ≈ −0.29 mol/(m^2^×h). Thus, the first option of selective separation is to set a current at which the resulting flux of the most mobile of the competing ions (Cl^−^) is zero. At the same time, H_2_PO_4_^−^ ions pass through the membrane at a relatively high rate, and the ion separation coefficient for H_2_PO_4_^−^ and Cl^−^ ions at this point reaches 12.5 (Figure 3b). However, as it was noted earlier, the value of this coefficient depends very strongly on the error in determining the concentration of ions, the resulting flux of which is zeroed. In addition, the change in concentration over time in the chambers of the EBM device is determined against the background of a high concentration of the analyte in the feed solution (0.05 M).

In the current range of 50 A/m^2^ < *i* < 100 A/m^2^, the second option of optimal selective separation can be chosen. Here, the resulting flux of a less mobile ion is negative (controlled by convective transport), and the flux of a more mobile ion is positive (controlled by electromigration). In other words, it is possible to simultaneously enrich one solution with a more mobile ion and the second solution with a less mobile one. In this case, it makes no sense to evaluate the separation selectivity by the $S_{Cl^{-}/H_{2}PO_{4}{}^{-}}$ or $S_{H_{2}PO_{4}{}^{-}/Cl^{-}}$ value. Only the absolute values of the fluxes of separated ions matter: The larger they are, the more efficient the separation process is. At 75 A/m^2^ (Figure 3b), it can be expected that the H_2_PO_4_^−^ flux of about −0.1 mol/(m^2^×h) and the Cl^−^ flux of about 0.27 mol/(m^2^×h) pass through the track-etched membrane. It should be noted that this interesting case, when the fluxes of separated ions are oppositely directed, cannot be realized by other membrane methods. Taking into account that only Cl^−^ ions can leave the feed solution, i.e., the outgoing fluxes of the competing H_2_PO_4_^−^ ions are zero, the separation coefficient should formally be set to infinity.

At 100 A/m^2^, the third possible option of selective separation of Cl^−^ and H_2_PO_4_^−^ ions is observed. Now the resulting flux of H_2_PO_4_^−^ ions is close to zero, $j_{H_{2}PO_{4}^{-}}$ = −0.055 mol/(m^2^×h). The flux of Cl^−^ ions is controlled by electromigration from chamber I to chamber II. Nominally, the ion separation coefficient, $S_{Cl^{-}/H_{2}PO_{4}{}^{-}}$, is −7.5, but since the separated ions are transported in different directions, the efficiency of such a process can hardly be overestimated. However, the value of the set electric current in the system is close to the limit of applicability, i.e., to the limiting current at the auxiliary membranes. In addition, the energy consumption in comparison with the first option of separation at 50 A/m^2^ increases significantly (about 0.50 and 0.67 kWh/mol Cl^−^ for the cell entirely).

The choice of one or another option of selective separation, in our opinion, depends on the concentration of the target and competing ions in the feed solution. It is less energy-consuming to remove the component, whose concentration is lower, from the solution. Probably, the first option will be more preferable for processing wastewater, since the concentration of H_2_PO_4_^−^ is almost always much lower than Cl^−^. This option should also be preferable when other anions besides Cl^−^ are present in wastewater, since the mobility of H_2_PO_4_^−^ is usually the lowest of all anions in wastewater, with the exception of organic compounds.

The values of the transport numbers, ${\tilde{t}}_{k}$, of competing anions were estimated by fitting the theory to the experimental data. As Equations (3)–(5) show, by changing ${\tilde{t}}_{k}$, the theoretical straight lines are shifted up or down when treating the *j*_k_ vs. ∆*p* data (at *i* = const) (Figure 3a); the slope is determined by the known values entering the expression $c_{k}\gamma d^{2}/(\eta L)$. When treating the *j*_k_ vs. *i* data (at ∆*p* = const) (Figure 3b), the slope of the theoretical straight lines is changed. The pore diameter value of 32 nm determined by the Hagen–Poiseuille equation (Equation (5)) from the experimental hydraulic permeability was used instead of the average diameter of 35 ± 3.0 nm determined by the SEM.

The best fit of the *j_i_* vs. ∆*p* data (at *i* = 25 A/m^2^) (Figure 3a) gives ${\tilde{t}}_{Cl^{-}}=0.55$ and ${\tilde{t}}_{H_{2}PO_{4}^{-}}=0.39$, while the fit of the *j*_i_ vs. *i* data (at ∆*p* = 0.3 bar) (Figure 3b) provides ${\tilde{t}}_{Cl^{-}}=0.32$ and ${\tilde{t}}_{H_{2}PO_{4}^{-}}=0.18$. A significant difference in the values of the ion transport numbers determined from two different lots of data is apparently associated with experimental errors, in particular, with a measurement error of ∆*p*. However, the averaged values of ${\tilde{t}}_{k}$ from these two lots are higher than the transport numbers in free solution ($t_{Cl^{-}}=0.36$ and $t_{H_{2}PO_{4}^{-}}=0.16$) determined from a simple relationship: $t_{k}=z_{k}^{2}D_{k}c_{k}/\sum_{j=1,2,3}z_{j}^{2}D_{j}c_{j}$. The average transport number of Na^+^ found by fitting is about 0.26 which is essentially lower than its value in the free solution, $t_{Na^{+}}=0.48$.

**Figure 4:**
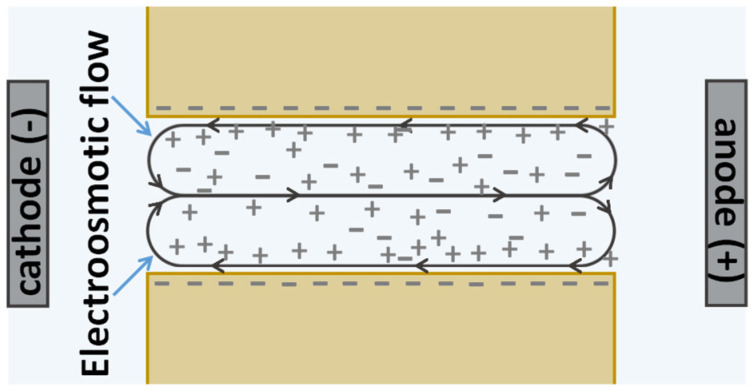
A scheme of induced convective flows in a membrane pore with negatively charged walls, when the membrane is located between two polarizing electrodes.

Hydroxyl and carboxyl groups are formed on the surface of the polymer (polyethylene terephthalate) after etching the tracks of TEM#811 [54]. These fixed groups form a negative electrical charge of the external surface and the pore walls at pH ≈ 4 [55], at which the experiments were carried out. This charge has a density of about 0.3 μC/cm^2^ according to Sabbatovskiy et al. [56]. Therefore, cations are concentrated near the pore walls. When an electric field is applied, electroosmosis occurs in the pores: The cations entrain the liquid along the pore walls in the direction from the anode to the cathode (Figure 4).

If no pressure difference is initially applied across the membrane, the fluid transported by electroosmosis will create excessive pressure in the cathode chamber and an induced pressure difference will appear. This induced pressure difference will cause the return flow in the opposite direction (from the cathode to the anode) in the central part of the pores (Figure 4). This return flow can entrain the anions and thus enhance their migration velocity, resulting in an apparent increase in their transport numbers. The effect is in a certain sense the opposite of the effect of inhibition of the forced convective transport of ions in a pore with charged walls described by Tang et al. [57]. This inhibition is due to electromigration caused by the streaming potential induced by the forced flow.

### 3.2. Anodic Alumina Membrane

The porous anodic alumina membrane (PAAM) is less permeable than TEM #811. Its measured hydraulic permeability is half that of TEM #811 (Table 2). In this regard, when separating Cl^−^ and H_2_PO_4_^−^ ions using PAAM, the current value of 50 A/m^2^ was fixed and the pressure drop was varied.

**Figure 5:**
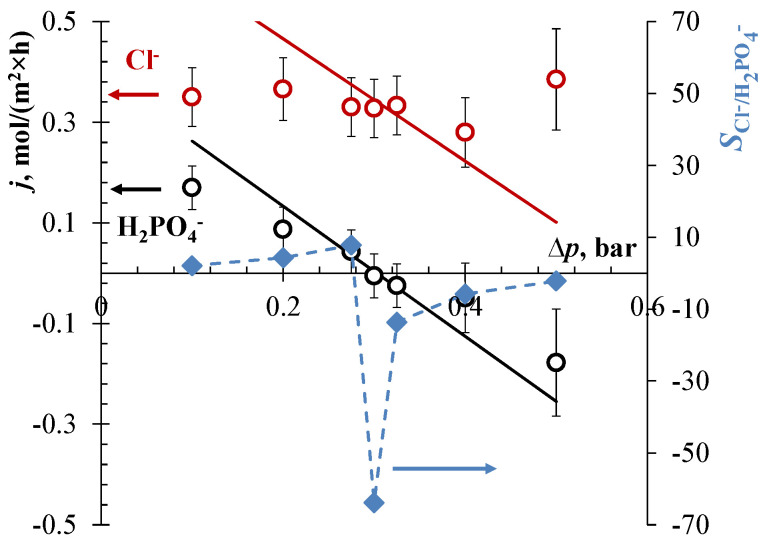
Flux densities of Cl^−^ and H_2_PO_4_^−^ ions (circles) through the PAAM membrane, as well as the separation coefficient, *S* (diamonds), vs. pressure drop (∆*p*) in the EBM system at a constant current density of 50 A/m^2^. Experimental data are shown by markers, and solid lines are calculated using Equations (3)–(5) with *d* = 38 nm, ${\tilde{t}}_{Cl^{-}}$ = 0.38, and ${\tilde{t}}_{H_{2}PO_{4}^{-}}$ = 0.21 as fitting parameters; the other membrane parameters are taken from Table 2.

Figure 5 shows that the varying pressure drop has almost no effect on the flux of chlorides through the PAAM. Obviously, the chloride flux will reach zero at a pressure drop outside the investigated range. However, it has been experimentally established that at a pressure drop above 0.5 bar, PAAM cannot be used, since the risk of its destruction increases significantly. Above 0.6 bar, the membrane cracks.

It was found that at a constant current density of 50 A/m^2^ and a pressure drop of 0.3 bar, the flux of H_2_PO_4_^−^ ions is close to zero. Within the measurement error, $j_{H_{2}PO_{4}{}^{-}}\approx$ −0.005 mol/(m^2^×h), while $j_{Cl^{-}}\approx$0.33 mol/(m^2^×h). A high separation selectivity ($S_{Cl^{-}/H_{2}PO_{4}{}^{-}}$= −66) can be achieved. However, the logical value of this coefficient tends to infinity, since the fluxes of separated ions are directed in the opposite direction, as in the case of using TEM #811 at currents of 75 and 100 A/m^2^.

It is known from earlier works that the porous anodic alumina membrane should exhibit anion-exchange properties at pH ≈ 3.9 of the feed solution [58,59]. In this regard, higher ${\tilde{t}}_{k}$ values were expected for both competing anions than were obtained by fitting (${\tilde{t}}_{Cl^{-}}$= 0.38 and ${\tilde{t}}_{H_{2}PO_{4}^{-}}$= 0.21), taking also into account the results for the TEM #811 membrane. The pore diameter value of 38 nm, determined by the Hagen–Poiseuille equation (Equation (5)) from the experimental hydraulic permeability, was used to fitting the transport numbers.

There are two reasons for these low transport numbers. First, although the pore walls are charged, the pore diameter is relatively large compared to the size of the separated ions. Hence, the transport numbers should be close to those in the free solution. Second, the stability of the PAAM properties during long-term operation is rather low. After the pretreatment of the membrane and pre-used with pressure to flush particles out of the etched channels, its hydraulic permeability is still not constant. Apparently, this causes a specific shape of the $j_{Cl^{-}}$ vs. ∆*p* dependence, and this complicates the correct determination of the parameters for the separation of Cl^−^ and H_2_PO_4_^−^ ions.

**Figure 6:**
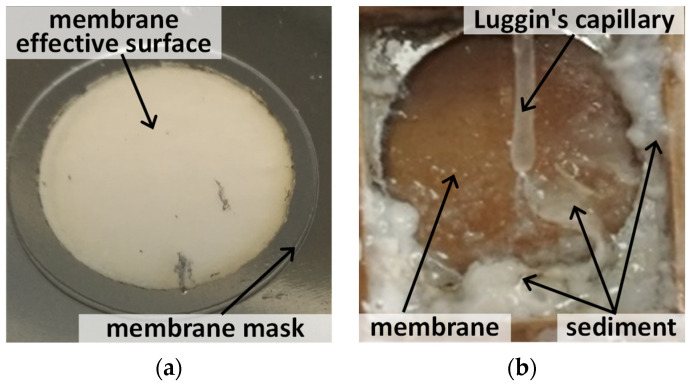
Photo of the surface of the (**a**) pristine PAAM and (**b**) operated for 20 h.

Under experimental conditions (pH = 3.8–3.9), the protonation of polymerized polyhydroxocomplexes of aluminum oxide, [Al_13_O_4_(OH)_24_(H_2_O)_12_]^7+^, constituting the matrix of the PAAM membrane, occurs. It causes anion-exchange properties of the membrane. However, during operation, the porous alumina membrane begins to degrade by the dissolution of water-soluble polyhydroxocomplexes and their further redeposition in the form of aluminum hydroxide [Al(OH)_4_]^−^ [45] (Figure 6). This leads to the clogging of pores, a decrease in hydraulic permeability, and a decrease in the fluxes of Cl^−^ and H_2_PO_4_^−^ ions to be separated. However, possible methods have been reported for making alumina membranes that are stable in aqueous solutions [60,61,62].

Thus, the EBM separation method can be effectively used for the selective separation of Cl^−^ and H_2_PO_4_^−^ singly charged anions. Despite the limitations associated with the allowable range of the currents, high selectivity can be achieved both when using the track-etched membrane and the membrane from porous alumina. However, in the latter case, there are additional limitations associated with the stability of the membrane characteristics.

Let us make a brief analysis of the obtained separation characteristics and compare them with similar characteristics found by other membrane methods. Table 3 presents the results from some recent papers on the selective recovery of phosphates in the presence of other anions using membrane technologies.

The fluxes of phosphoric acid ions through the membrane, as well as the fluxes of competing anions, were calculated using the published data presented in the relevant articles [22,63,64,65,66,67,68]. Calculations were made using Equations (1) and (2).

It is known that selective electrodialysis or selectrodialysis (S-ED), using special-grade monovalent-ion-selective ion-exchange membranes, makes it possible to successfully separate monovalent and multivalent ions of the same charge sign [49,69]. Using conventional ED with monopolar single-layer ion-exchange membranes, it is possible to concentrate certain types of ions. Rotta et al. [63] reported that during an ED process, the concentration of H_x_PO_4_^(3-x)−^ ions in the concentration chamber increased by about 10 times. However, the concentration of the competing SO_4_^2−^ ions increased approximately by the same factor. The low selectivity of the used anion-exchange membrane for doubly charged SO_4_^2−^ ions ($S_{H_{x}PO_{4}^{(3-x)-}/SO_{4}^{2-}}$ = 0.64) is explained by electrostatic interactions [70] and takes place only at low current densities [71]. The performance of the conventional ED depends on the applied current/voltage [64] and is limited due to the pH variation in the ED chambers.

Bipolar membrane electrodialysis (BMED) typically also uses monopolar ion-exchange membranes for separation [65]. At the same time, the use of bipolar membranes makes it possible to generate H^+^ and OH^−^ ions without reagents and to obtain different products with relatively high productivity in separated chambers. For example, a reagentless pH shift allows selective extraction of N^III^ and P^V^ from the feed solution in the form of NH_4_^+^ ions and H_2_PO_4_^−^ ions. In this mode, the NH_3_ was concentrated up to 16 g/L in the base solution [65].

S-ED uses monovalent-ion permselective IEMs, which help to solve the problem of separation of ions of the same charge sign [66]. Neosepta AMS, CMS, ACS and CIMS (Astom Corp., Shunan, Japan); Selemion ASV and CSO (AGC Engineering Co., Ltd., Chiba, Japan); Fumasep FAA, FKL and FKE (FuMA-Tech, Bietigheim-Bissingen, Germany) are among commercial membranes of this special grade. They pass singly charged ions but reject multiply charged ones. Table 3 presents our estimates of the selective separation parameters of HPO_4_^2−^, Cl^−^, and NO_3_^−^ ions using a special-grade Neosepta ACS membrane [66]. The technology allows selective separation of HPO_4_^2−^ from Cl^−^ and NO_3_^−^ ($S_{HPO_{4}{}^{2-}/Cl^{-}}$ = 0.09 and $S_{HPO_{4}{}^{2-}/NO_{3}^{-}}$ = 0.175). At the same time, $S_{Cl^{-}/NO_{3}^{-}}$ for Cl^−^ and NO_3_^−^ ions transferred through this membrane is 2. Therefore, selectrodialysis can be used to enrich the phosphate proportion even in the presence of high chloride concentrations [64,67]. The presence of competing anions in the feed solution has little effect on the selectivity coefficient, but significantly increases the solution processing time to the same degree as the phosphate removal [67].

As noted in the introduction, commercial nanofiltration membranes can also be used to concentrate H_x_PO_4_^(3-x)−^ ions due to the high retention rate [22,68]. Our estimates (Table 3) lead to the conclusion that nanofiltration can be effectively used both to separate ions of different charge values and for ions of the same charge value. For example, when separating singly charged H_2_PO_4_^−^ and Cl^−^ ions, the separation coefficient, $S_{Cl^{-}/H_{2}PO_{4}{}^{-}}$, can reach ~15–25 ($S_{H_{2}PO_{4}{}^{-}/Cl^{-}}$~0.04–0.07; $j_{Cl^{-}}$~0.57 mol/(m^2^×h), $j_{H_{2}PO_{4}^{-}}$~4.3 × 10^−3^ mol/(m^2^×h)) [22].

The hybrid EBM method used in this work, as well as nanofiltration, makes it possible to separate both ions of the same charge and different charge values, in contrast to the above-mentioned electromembrane (ED) methods of selective separation [3]. The EBM method is intensively studied now. In earlier papers, the effectiveness of the method has been proven in the separation of binary mixtures of Li^+^/Na^+^, Li^+^/K^+^, and Li^+^/Ca^2+^ ions for lithium extraction [38,39,40,41]. The researchers selected such separation parameters (current density and pressure drop) so that competing ions (Na^+^, K^+^, and Ca^2+^) were transported through the membrane, while lithium ions remained in the feed solution. The fluxes of Na^+^, K^+^, and Ca^2+^ were 0.28, 0.44, and 0.44 mol/(m^2^×h), and the separation coefficients $S_{Li^{+}/M^{n+}}$ were 0.35, 0.085, and 0.27, respectively. In the case of processing the Li^+^/Na^+^/K^+^ ternary mixture, the separation efficiency decreased by 1.5–2 times [36].

In recent works, the ion separation coefficient for the Li^+^/K^+^ pair can vary from 59 [32] to 150 [31,42]. When Li^+^/Na^+^ ions are separated, the selective permeability coefficient is somewhat lower and reaches 30 [31]. The flux of lithium through the membrane under optimal conditions can be ~0.5 mol/(m^2^×h) [3]. If lithium remains in the feed solution and the competing K^+^ ion is transferred through the membrane, as in the works of Konturri et al. [38,39,40,41], its flux ($j_{K^{+}}$) can be up to 2.1 mol/(m^2^×h) [3,32].

Of course, the EBM method has limitations, similar to all membrane methods. As discussed above, this is primarily due to the processes occurring at auxiliary ion-exchange membranes. However, the method demonstrates high performance for the separation of ionic components compared to other membrane methods (Table 3).

Generally, the application of membrane methods can significantly reduce or exclude the use of chemicals. Nowadays, in industry, ion separation is carried out using reagent-based technologies (hydrometallurgy). For example, by combining the EBM method with reverse osmosis, conventional and selective ED, it is possible to arrive at a technology for the reagentless extraction of valuable components [3].

## 4. Conclusions

In this work, the capability of the hybrid electrobaromembrane (EBM) method for the separation of singly charged Cl^−^ and H_2_PO_4_^−^ anions was studied. The EBM technology differs from other membrane methods where the separation occurs under the action of an electric field and pressure simultaneously. Separation selectivity is achieved due to the difference in the electrical mobility of the ions being separated. It has been shown that at least three options of separation of this pair are possible: to reduce the flux of the most mobile ion to zero, to reduce the flux of the less mobile ion to zero, or to organize the process so that the separated ions move through the porous membrane in different directions. In the latter case, the H_2_PO_4_^−^ flux of about −0.055 mol/(m^2^×h) and maximum Cl^−^ flux of about 0.40 mol/(m^2^×h) through a track-etched membrane can be expected at 0.3 bar and 100 A/m^2^. Dihydrogen phosphate ions can be removed from the solution at lower currents (50–55 A/m^2^), when their resulting flux is controlled by convective transport (−0.23–0.29 mol/(m^2^×h)), while the chloride flux through the membrane is close to zero. Efficient separation of these ions using a porous anodic alumina membrane is achieved with the same separation parameters (0.3 bar, 50 A/m^2^). Under these conditions, chloride flux can be estimated as 0.33 mol/(m^2^×h). The separation efficiency can be high when using both types of porous membranes due to the possibility of directing the fluxes of separated ions in opposite sides, which is unattainable when using other membrane methods.

## Figures and Tables

**Table 1:** Some characteristics of ions (at 25 °C) from the feed solution [43].

Ion	Symbol	Diffusion Coefficient, 10^−9^ m^2^/s	Stokes Radius, Å
Sodium	Na^+^	1.33	1.84
Dihydrogen phosphate	H_2_PO_4_^−^	0.88	2.79
Chloride	Cl^−^	2.03	1.21

**Table 2:** Some characteristics of the porous membranes used in this study.

Parameters	TEM #811	PAAM
Thickness	10 μm	80 μm
Pore density *	5.0 × 10^9^ pores/cm^2^	1.0 × 10^10^ pores/cm^2^
Pore diameter *	35 ± 3.0 nm	44 ± 2.0 nm
Surface porosity	5.3 ± 1.0%	16.7 ± 1.5%
Hydraulic permeability	0.10 ± 0.02 cm^3^/(cm^2^·min·bar)	0.06 ± 0.02 cm^3^/(cm^2^·min·bar)
Functional groups	Hydroxyl and carboxyl groups [44]	Alumina polyhydroxocomplexes [45]

* Estimated by scanning electron microscopy, SEM.

**Table 3:** Comparison of recovery/rejections of phosphates using different membrane methods.

Method	Membrane	Feed Solution	Experiment Details	$j_{H_{x}PO_{4}{}^{(3-x)-}},\;\mathrm{mol}/(m^{2}\times h)$	Competing Anion, A^−^	$j_{A^{-}},\;\mathrm{mol}/(m^{2}\times h)$	$S_{H_{x}PO_{4}{}^{(3-x)-}/A^{-}}$
Conventional Electrodialysis Ref. [63]	2 cell pair with HDX100 CEM and HDX200 AEM (Iontech, Hangzhou, China)	3.4 × 10^−3^ M Na_2_SO_4_,	7 A/m^2^	4.2 × 10^−3^	SO_4_^2−^	0.137	0.64
		8.2 × 10^−5^ M Na_2_HPO_4_·7H_2_O,					
		8.0 × 10^−5^ M NaH_2_PO_4_·H_2_O					
		(pH ≈ 7.0)					
Selective Electrodialysis Ref. [64]	5 cell pair with monovalent-selective PC-MVK and PC-MVA membranes, as well as PC-SA, PC-SK, PC-SC (PCA GmbH, Heusweiler, Germany)	1.3 × 10^−3^ M NaH_2_PO_4_·H_2_O,	8 V (~45 A/m^2^)	6.4 × 10^−3^	SO_4_^2−^	0.017	0.29
		0.036 M NH_4_Cl,					
		1 × 10^−3^ M Na_2_SO_4_,					
		0.01 M KCl,					
		2.5 × 10^−3^ M MgCl_2_,					
		2.5 × 10^−3^ M CaCl_2_					
		(pH = 4.9)					
Bipolar Membrane Electrodialysis Ref. [65]	5 cell pair with commercial heterogeneous CEM, AEM, and BPM (MemBrain^®^, Stráž pod Ralskem, Czech Republic)	0.08 M NH_4_Cl,	~167 A/m^2^, <60 V	0.144	Cl^−^ CH_3_COO^−^	0.46 0.35	1.2 1.3
		0.075 M (NH_4_)_2_SO_4_,					
		0.022 M NaH_2_PO_4_,					
		0.07 M CH_3_COONH_4_,					
		0.014 M H_3_PO_4_,					
		2.64 mL/L butyric acid,					
		2.04 mL/L valeric acid					
		(pH = 6.0)					
Selective Electrodialysis Ref. [66]	3 cell pair with monovalent-selective Neosepta ACS, as well as Neosepta CMX and Neosepta AMX (Astom Co., Shunan, Japan)	0.01 M NaCl,	9 V	1.2 × 10^−4^	Cl^−^ NO_3_^−^	0.043 4.5 × 10^−3^	0.09 0.175
		2.1 × 10^−3^ M NaNO_3_,					
		3.2 × 10^−4^ M Na_2_HPO_4_,					
		(pH = 7.0)					
Conventional Electrodialysis and Selective Electrodialysis Ref. [67]	3 cell pair with monovalent-selective PC-MVA, as well as PC-SA and PC-SK (PCA GmbH, Heusweiler, Germany)	0.023 M Cl−,	62.5 A/m^2^ <12 V	0.016 over the PC-MVA membrane, S-ED 0.036 over the PC-SA membrane, ED	Cl^−^	1.32 1.76	0.28 0.47
		1 × 10^−3^ M HxPO_4_^(3-x)−^,					
		2 × 10^−3^ M NO^3−^,					
		2 × 10^−3^ M HCO^3−^,					
		2 × 10^−3^ M SO_4_^2−^,					
		2 × 10^−3^ M Ca^2+^,					
		2 × 10^−3^ M Mg^2+^					
		(pH = 5.5)					
Nanofiltration Ref. [22]	NF270 membrane (Dupont, New York, NY, USA)	0.01 M NaCl,	20 bar	~0.9 × 10^−3^ for HPO_4_^2−^	Cl^−^	~0.57	~0.04- 0.07
		2 × 10^−3^ M K_2_HPO_4_		~4.3 × 10^−3^ for H_x_PO_4_^−^			
		(pH = 8.9)					
	
		0.01 M Na_2_SO_4_,		~0.3 × 10^−3^ for HPO_4_^2−^	SO_4_^2−^	~3.2 × 10^−3^	0.7−10
		0.5 × 10^−3^ M K_2_HPO_4_		~1.5 × 10^−3^ for H_x_PO_4_^−^			
		(pH = 7.2)					
Nanofiltration Ref. [68]	NF200 (Dow, Midland, MI, USA)	1 × 10^−3^ M NaCl,	5 bar	2.8 × 10^−3^	Cl^−^ NO_3_^−^	9.5 × 10^−3^ 0.036	0.5 0.25
		1 × 10^−3^ M NaNO_3_,					
		1 × 10^−3^ M NaH_2_PO_4_					
		(pH = 5.2–5.4)					
Hybrid Electrobaromembrane (EBM) method [this work]	1 cell pair with TEM #811 track-etched membrane	0.05 M NaCl, 0.05 M NaH_2_PO_4_ (pH = 3.8–3.9)	0.3 bar, 50 A/m^2^	−0.285	Cl^−^	−0.022	12.5
			0.3 bar, 100 A/m^2^	−0.055		0.402	−0.13
	or porous anodic alumina membrane (PAAM)		0.3 bar, 50 A/m^2^	−0.005	Cl^−^	0.330	−0.015
	Both porous membranes were supplemented by two MK-40 (JCC Shchekinoazot, Russia)						

## Data Availability

Not applicable.
